# Religious Opposition to Polio Vaccination

**DOI:** 10.3201/eid1506.090087

**Published:** 2009-06

**Authors:** Haider J. Warraich

**Affiliations:** Aga Khan University Medical College, Karachi, Pakistan

**Keywords:** Poliomyelitis, poliovirus, immunization, Pakistan, Afghanistan, Nigeria, Islam, religious opposition, viruses, letter

**To the Editor:** In 1988, the Global Polio Eradication Initiative was formed, with the aim of reducing infection with poliomyelitis virus. Two decades later in 2008, a total of 1,625 children contracted acute flaccid paralysis caused by poliovirus infection (*1*). This finding represented a 150% increase over the number of cases in 2007 (*1*) and resulted in the reemergence of polio as one of the world’s deadliest infections. As of 2009, polio remains endemic to 4 countries (India, Nigeria, Pakistan, and Afghanistan); in 2008, cases were also detected in 14 other countries.

Religious opposition by Muslim fundamentalists is a major factor in the failure of immunization programs against polio in Nigeria (*2*), Pakistan (*3*) and Afghanistan (*4*). This religious conflict in the tribal areas of Pakistan is one of the biggest hindrances to effective polio vaccination. Epidemiologists have detected transmission of wild poliovirus from polio-endemic districts in Afghanistan, most of which are located in the southern region of this country bordering Pakistan, to tribal areas of Pakistan (*4*). This transmission has resulted in new cases of polio in previously polio-free districts. The local Taliban have issued *fatwas* denouncing vaccination as an American ploy to sterilize Muslim populations. Another common superstition spread by extremists is that vaccination is an attempt to avert the will of Allah. The Taliban have assassinated vaccination officials, including Abdul Ghani Marwat, who was the head of the government’s vaccination campaign in Bajaur Agency in the Pakistani tribal areas, on his way back from meeting a religious cleric (*5*). Over the past year, several kidnappings and beatings of vaccinators have been reported. Vaccination campaigns in Nigeria and Afghanistan have also been hampered by Islamic extremists, especially in the Nigerian province of Kano in 2003, which has resulted in the infection returning to 8 previously polio-free countries in Africa (*2*).

Before the Global Polio Eradication Initiative in 1988, a total of 1,000 persons/day were infected with a virus that would cripple them for the rest of their lives (*6*). To eradicate the disease, 1 major factor will be to gain support of those susceptible to fundamentalist propaganda. Islam is a progressive religion, and religious leaders should be asked to support polio eradication programs. The Imam of the Ka’aba and other influential religious figures should be asked to highlight the plight of children with polio. Vaccinators operating in conflict-ridden areas should be provided protection so that they are better able to perform their duties. Not only will children in these areas be safer, but the disease will not be exported to areas where wild polio transmission has been interrupted by vaccination. Further study of the attitudes of Muslim populations toward vaccination is needed.
